# Scalable and Cost-Effective Assignment of Mobile Crowdsensing Tasks Based on Profiling Trends and Prediction: The ParticipAct Living Lab Experience

**DOI:** 10.3390/s150818613

**Published:** 2015-07-30

**Authors:** Paolo Bellavista, Antonio Corradi, Luca Foschini, Raffaele Ianniello

**Affiliations:** Dipartimento di Informatica—Scienza e Ingegneria (DISI), University of Bologna, Viale Risorgimento, 2, 40126 Bologna, Italy; E-Mails: paolo.bellavista@unibo.it (P.B.); antonio.corradi@unibo.it (A.C.); raffaele.ianniello@unibo.it (R.I.)

**Keywords:** mobile crowd sensing, mobile crowd datasets, smart cities, task assignment strategies, performance evaluation

## Abstract

Nowadays, sensor-rich smartphones potentially enable the harvesting of huge amounts of valuable sensing data in urban environments, by opportunistically involving citizens to play the role of mobile virtual sensors to cover Smart City areas of interest. This paper proposes an in-depth study of the challenging technical issues related to the efficient assignment of Mobile Crowd Sensing (MCS) data collection tasks to volunteers in a crowdsensing campaign. In particular, the paper originally describes how to increase the effectiveness of the proposed sensing campaigns through the inclusion of several new facilities, including accurate participant selection algorithms able to profile and predict user mobility patterns, gaming techniques, and timely geo-notification. The reported results show the feasibility of exploiting profiling trends/prediction techniques from volunteers’ behavior; moreover, they quantitatively compare different MCS task assignment strategies based on large-scale and real MCS data campaigns run in the ParticipAct living lab, an ongoing MCS real-world experiment that involved more than 170 students of the University of Bologna for more than one year.

## 1. Introduction

The large availability of mobile devices with sensing capabilities, combined with the pervasive spread of communication infrastructures, gave rise, in recent years, to a number of platforms for Mobile Crowd Sensing (MCS). MCS is commonly referred to as a paradigm for distributed gathering of heterogeneous sensing data from pocket devices used by the crowds. In fact, the spontaneous widespread diffusion of Internet-connected sensor-equipped devices has enabled one to accurately trace world-related information and (physical) activities of citizens, by taking advantage of people willing to collaborate toward a continuous data harvesting process, which is feasible and highly effective, in particular, in smart cities areas where people density is high and potential participants almost constantly bring their smartphones [1]. MCS solutions could be of extreme importance from the community/smart city managers perspective because they can enable the monitoring of areas that are still not covered by fixed monitoring infrastructures (noise pollution sensors, surveillance cameras, *etc.*). In addition, MCS may benefit from being citizen-centric, in the sense that it allows getting not only raw and locally processed sensing data but also (geo-located) constructive comments and suggestions by citizens. Moreover, MCS generally fosters a sense of participation because it allows people to be and feel active in the smart city monitoring-and-management loop, volunteering for the execution of given sensing activities (called *tasks* in the following) but also actively participating through their actions, which may impact and modify the physical world eventually (citizens as monitors but also *actuators* in the smart city). Finally, as recognized also by all major smartphone vendors, which are very recently starting to propose activity/geolocation sensing apps as part of their default distributions, sensed data represent an invaluable information big data depot, in order to foster the design and development of new and unforeseen services based on them.

Motivated by this continuously increasing trend, several MCS platforms appeared to facilitate the management of the whole MCS workflow. Indeed, employing users and devices to collect data from the real world poses significant social and technical challenges. From the social perspective, it is crucial to motivate users to participate, for example, by providing useful crowdsensing-based services, handing out incentives, and fostering a sense of participation in a community [2,3]. It is important not to overload users with duties over the limit of what they can and feel to contribute in crowdsensing, and to keep them involved by avoiding that the MCS process becomes too repetitive, boring, or even annoying for them. From a technical point of view, it is of paramount relevance that sensing software running on user devices does not negatively impact on user quality of experience, with negative consequences on the quantity and quality of collected data. In any case, the boundary between social and technical challenges is not clear cut: for example, the minimization of the global resource overhead by considering a minimal subset of users in a crowdsensing campaign requires also analyzing geo-social profiles, identifying and inferring which users are most likely to successfully harvest the required data. These differentiated MCS strategies and policies, toward overall MCS optimization, are very important; however, while some first efforts in the past have addressed these issues with some initial theoretic discussions and simulative approaches [4], only a few seminal efforts used real and over-the-city MCS living labs, with the goal to draw consistent guidelines and lessons about the feasibility and effectiveness of employed optimization solutions in real MCS testbeds.

The ParticipAct project and living lab addresses all these issues with the overall goal of enabling a new class of MCS systems as large-scale testbeds usable by the many interdisciplinary research communities working on smart cities. Within the ParticipAct project, we have already addressed several related issues, which span from smartphone sensing and user activity detection [5] to enabling geo-localized tasks [6], from enabling people participation [7] to designing the overall architecture and living lab [8]. This paper makes an innovative and relevant step further by originally focusing on increasing the effectiveness of the proposed MCS campaigns along two primary solution guidelines. On the one hand, we propose and thoroughly assess different task assignment policies based on profiling and prediction of user mobility patterns, by also considering how the location and time of user notification influences the rates of task acceptance and completion. On the other hand, we introduce new facilities to gain higher user participation: (i) task co-creation, to let participants (and not only administrators/smart city managers) create and propose their own MCS tasks; and (ii) gamification, to increase user involvement through competition and virtual incentives. In addition, the paper reports a thorough performance assessment of the related and newly available platform facilities, by discussing and evaluating their strength and weaknesses, and drawing some important suggestions of general applicability and validity for the community of researchers working in this field. Finally, ParticipAct is available as an open-source platform that allows for fast development and deployment of large-scale MCS experiments with minimal intrusion and resource usage on both smartphone and server sides at http://participact.unibo.it/.

The rest of the paper is structured as follows: Section 2 overviews the main project motivations and architecture design guidelines, while Section 3 describes the overall ParticipAct architecture and Section 4 delves into the details of the new gamification and co-creation facilities. Large space is devoted to Section 5 that reports a thorough evaluation, with experimental results collected during the first 16 months of MCS campaigns in the city of Bologna. An analysis of related work (Section 6) and an assessment of the current state of ParticipAct as well as of its future goals conclude the article.

## 2. ParticipAct Goals and Design Guidelines

ParticipAct has the primary goal of realizing an efficient and easy-to-use playground in which to verify, practically in actual in-the-field deployment environments, any desired MCS strategy, as well as to identify the most suitable MCS policies depending on context and application requirements. The research goals of ParticipAct are manifold:

- designing and testing a generic MCS architecture;
- evaluating, on a large scale, scalable solutions for classifiers and machine-learning-based algorithms, specifically fitting MCS goals and requirements;
- assessing the challenges of managing human resources involved in MCS;
- maintaining high interest in participants in order to keep them active and involved.

To pursue these goals, the ParticipAct experiment engages about 170 smartphone-provided students for more than one year of active participation: enrolled students, selected to provide a statistically relevant group of heterogeneous individuals, can passively collect data by their smartphones but can also be asked to perform requested activities, typically within their usual localities of physical presence. In any case, they can voluntarily decide to either accept or refuse to collaborate toward the requested MCS actions, and they can also freely stop and abandon them at any time.

ParticipAct is the complete supporting infrastructure that allows local actions and the client collection of data, is in charge of transferring sensed data to the ParticipAct back-end support, and takes over not only data harvesting, but also post-processing, mining, and maintenance. ParticipAct has already attracted much external interest and catalyzed other independent and parallel experiments, mainly within the city area of Bologna but also for larger communities. That has motivated us to organize the ParticipAct platform and its main experiment as a backbone for MCS, with the possibility to easily add and compose additional sensing activities and possibly to schedule and launch new related MCS campaigns. By considering in the ParticipAct architecture and the experiences already made with our platform, they have allowed us to define some distilled guidelines and principles to be taken into account in MCS campaigns and supports:

- *Minimal intrusion on client devices*—smartphone computing overhead must be minimized in both active actions (e.g., completing a requested via explicit operations from users carrying the sensing smartphones) and passive actions (e.g., smartphone-based monitoring of cyber-physical indicators without direct involvement of the carrying users), as well as in local and distributed transmission phases.
- *Fast feedback and minimal delay in producing stream information*—data must be quickly provided according to application- or deployment-specific policies that can be tuned and agreed on with policy administrators and controllers.
- *Openness and security*—an MCS platform should share (a portion of or the whole) sensed data with other companion experiments within a precise security boundary, which enables sharing with and only with authorized apps.
- *Complete data management workflow*—an MCS platform should support any step in the sensed data management cycle, from collection to communication, from post-processing and maintenance to mining and result provisioning.
- *Users’ involvement through gamification*—users need to be continuously stimulated with proper incentives in order to avoid individual or group effects of interest decrease, loss of active participation, responsiveness degradation, *etc*.

These features, better described in the following, together with technical details about their effective and efficient implementation, make ParticipAct a complete MCS platform, which encompasses the whole process from data collection to post-processing and mining. Moreover, ParticipAct contributes to the field by being available to the MCS community as an open source project to be used, refined, and extended.

## 3. ParticipAct: Model and Architecture

Smartphones are nowadays powerful and ubiquitously available sensors for data gathering in smart city scenarios. At the same time, in order to make MCS practically viable and effective in real wide-scale scenarios, it is necessary to have the possibility to orchestrate the whole MCS workflow, as pointed out in the previous section. In other words, common goals of any MCS platform should be to include efficient capabilities of orchestrating crowdsensing campaigns, by delivering sensing tasks to the most promising citizens (the ones who are more likely to complete the assigned tasks), and of collecting sensing data by storing and processing them to better manage and control next campaigns. This section overviews the ParticipAct platform, which aims to make a step further in MCS by enabling a long and still running living lab that allows one not only to assess the MCS platform from a technological viewpoint but also to quantify its effectiveness in collecting data and in keeping people involved in the MCS loop.

The ParticipAct platform, similarly to other MCS platforms, adopts a client-server architecture that includes a client, running on user devices to manage tasks and to run all required sensing activities by interacting with participants via their smartphones, and a server to store, process, and present collected results [9]. The ParticipAct client takes care of receiving tasks, asking users whether they want to run them, managing data collection, and uploading results. The ParticipAct server, instead, provides advanced management, storage, and analysis features for crowd-sensed data and consists of two main parts: the back-end that takes care of receiving, storing, and processing sensed data; and the crowdsensing manager that provides the administration interface to design, assign, and deploy sensing tasks. Finally, at the core of ParticipAct MCS campaigns, there are tasks, defined as fine-grained simple operations that are more likely to be completed by users. Section 3.1, Section 3.2 and Section 3.3 describe, respectively, the ParticipAct task model, client, and server architecture extensively.

### 3.1. Task Model

A task is a representation of a crowdsensing campaign to gather sensing indicators (considered relevant) at the desired quality level and with the desired coverage. In ParticipAct a task is modeled in terms of five fundamental components:

- *description*: a textual/graphical representation, prompted to users, of what to do to complete the task;
- *duration*: time span granted to a user in order to complete a given task, after having explicitly accepted it;
- *acceptance window*: time boundaries for a user to accept a given task;
- *associated points*: points granted to users who complete a given task successfully;
- *geographic area associated with the task*: geographic boundaries inside which users can receive notification of the new task assignment and execute it successfully.

**Figure 1:**
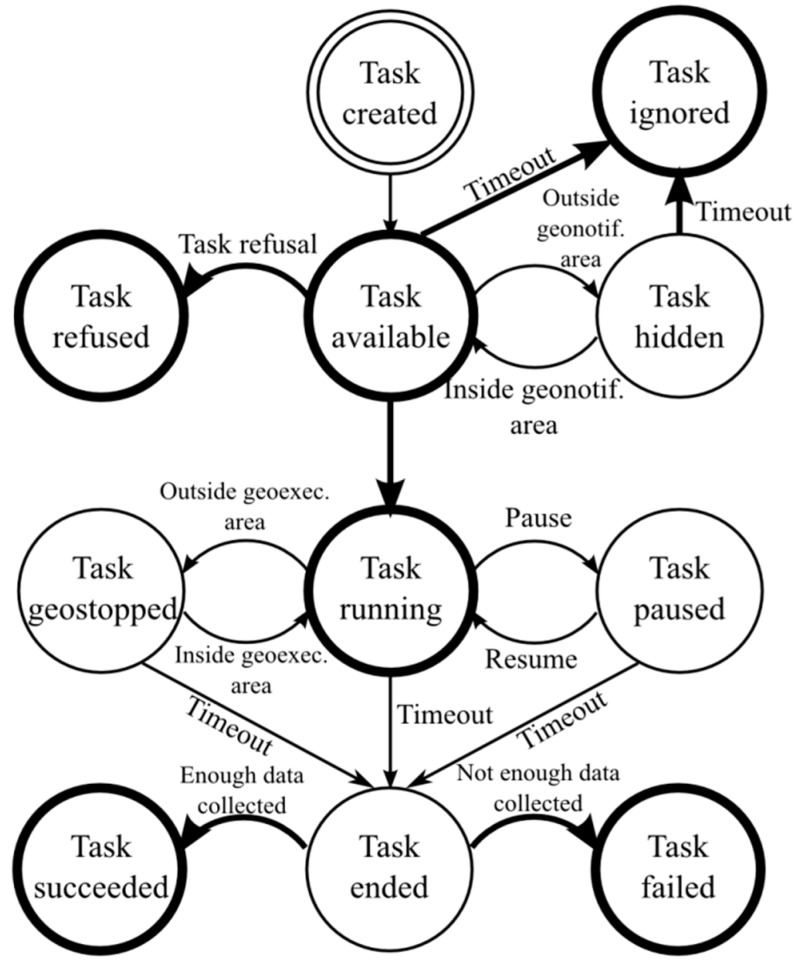
Task state lifecycle. Data collection is enabled only in the running state. States with a bold stroke reached through transitions represented with a bold arrow are kept in sync between clients and the server.

In addition, any task is associated with a set of actions (either active or passive) to complete. During its life, a task can reach different states. Figure 1 shows the lifecycle of a ParticipAct task. The initial state is the creation of the task. After creation, the task passes to a state where it is listed as available. The task moves from the available state according to the transitions below:

- if the task is rejected by the user, then it transits to refused state;
- after a period of time greater than the acceptance window configured for that task, the task transits into ignored state;
- if the task is accepted by the user, then it transits into execution state;
- if the task is geo-localized, then, at first, it passes to a hidden state to prevent the user to accept/reject it and, then, when the associated user traverses the area of interest, it becomes available. If, during the period in which the state is hidden, the acceptance window expires, then it passes directly to the ignored state.

During sensing data collection, execution may be stopped for two main reasons: either because the user decides to pause voluntarily collecting the data or, if the task is geo-localized, when the user exits from the area of interest. In any case, from each of these situations the task transits to a final state when the associated task duration ends. The task will then be evaluated (either successful or failed) by the ParticipAct platform based on the associated data that were collected.

As we can see from Figure 1, there is not a strict and expensive synchronization between clients and the server. In fact, to keep task client/server state perfectly synchronized, state changes should be allowed anytime clients can communicate them to the server, thus making state transitions very costly because network availability on mobile devices is often spotty. Conversely, some state changes do not need to be synchronized between clients and the server infrastructure because they are temporarily useful only on the client side (e.g., when a task is temporarily paused). That is the motivation of the state machine in Figure 1: only relevant task state transitions call for synchronization; in particular, transitions to available, refused, ignored, running, succeeded, and failed states are transparently synchronized with the server in the ParticipAct platform; if one of those transitions occurs when there is no data connectivity, the task state is implemented via a soft state to be automatically finalized as soon as the server acknowledges it.

### 3.2. The ParticipAct Client Architecture

**Figure 2:**
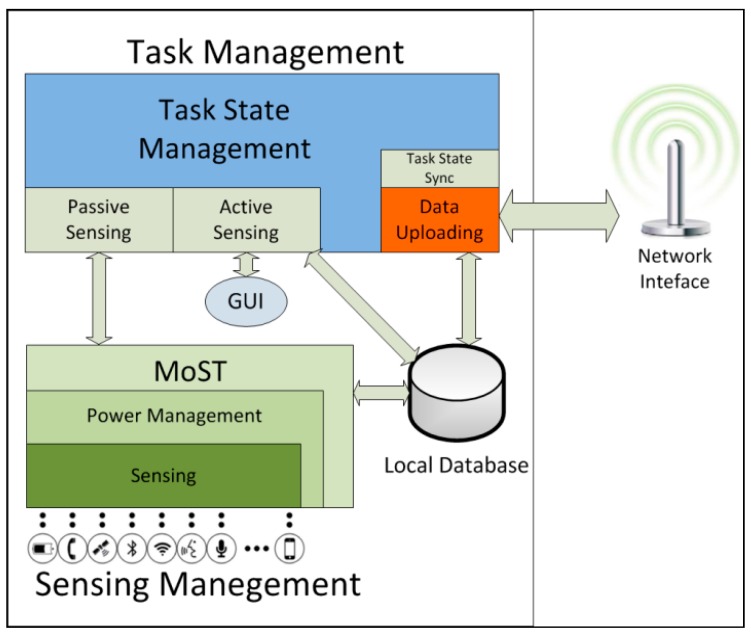
The architecture of the ParticipAct client.

The ParticipAct client is the component in charge of receiving tasks, asking users whether they are willing to run them, managing data collection, and uploading results. The client allows an easy data collection from the user point of view: tasks are presented to users on their smartphone displays and data collection starts when the task is explicitly accepted, by requiring minimum user intervention. Functionally, the ParticipAct client consists of two macro-components: the task management one and the sensing management one (Figure 2). These components orchestrate the full lifecycle of tasks on user devices and are responsible for both interacting with users and efficiently accessing smartphone sensors.

#### 3.2.1. Task Management

The task management macro-component is responsible for managing crowdsensing campaigns by delegating actions to active and passive sensing modules. It also takes care of overseeing the whole task lifecycle on smartphones. It has five main responsibilities realized by its components: (i) receiving tasks from the server and keeping their state synchronized; (ii) providing users with an interface to control task execution; (iii) implementing the Graphical User Interface (GUI) for active sensing actions with user interaction; (iv) commanding and managing sensing actions; and (v) uploading sensed data.

The Task State Sync and Task State Management components are in charge of the first duty, by taking care of receiving new tasks and, in acceptance, by driving their full lifecycle. As stated previously, only important state transitions are communicated to the server and they occur only if the server acknowledges them. In addition, the Task State Management component gives users the opportunity of completely controlling the sensing process: whenever an available task is pushed to user devices, the task management component gives users the opportunity to either accept or refuse it, thus allowing different dynamic levels of engagement by different users in the managed MCS campaigns.

The GUI component enables sensing actions that require active user participation. Currently, ParticipAct supports four active sensing actions that allow to collect data available only through explicit user collaboration, thus enabling MCS scenarios such as collaborative journalism, urban photographic mapping, and geotagging. In particular, the supported active sensing actions are surveying, taking a picture, tagging a place, and moving to a place in a given time window. ParticipAct implements a custom GUI for any of them.

Task Management also drives sensing actions: it starts/stops passive actions based on the current state of the task, *i.e.*, when the user pauses data collection or when the user moves outside the target area in the case of geo-executed tasks. Actual activation/deactivation of sensors is demanded to the Passive Sensing component that sends appropriate requests to the Sensing Management macro-component.

Finally, the Data Uploading component is in charge of retrieving sensed data and uploading them on the server. This process has to balance between uploading data as soon as possible to the server and minimizing the associated power consumed by radio interfaces. According to the minimal intrusion principle, ParticipAct data upload is geared towards minimizing its impact on battery lifetime. To accomplish this, ParticipAct batches data uploads and requires a minimum interval of five minutes (configurable to different values anyway) between two consecutive uploads, which grants the radio interface enough standby time [10]. Local Database temporarily stores data until the server acknowledges their reception, thus guaranteeing no data loss even in the presence of unreliable data connections and client device shutdown.

#### 3.2.2. Sensing Management

The Sensing Management component plays a pivotal role in ParticipAct crowdsensing because it manages the access to all sensors available on smartphones and the collection and processing of their output. Sensing is a power-hungry process that should be carefully driven to avoid negative impact on users, again following the minimal intrusion principle, which is very relevant for practical adoption of MCS platforms. About sensing management, in ParticipAct we have distilled three design guidelines. First, sensing should promote availability of high-level inferences, meaning that while accessing sensors on a smartphone (e.g., accelerometer) is a relatively trivial process, providing high-level inferences (e.g., the user is walking/running/standing) is a much more valuable feature. Second, sensing should be resource-aware: sensing management should put effort in minimization of resource consumption to both reduce impact on battery lifetime and limit performance degradation effects on user devices. Third, sensing should be system-aware: the sensing system coexists with the runtime OS support and the other executing applications; with them, it competes for un-shareable resources (e.g., microphone and camera can be used only by one application at a given time); the sensing system should be able to transparently resolve conflicts and to promote a non-intrusive approach.

These principles drove us to the development of our original sensing system, called Mobile Sensing Technology (MoST). MoST is our open-source Android sensing library that provides a uniform access layer to all physical and logical sensors, thus relevantly simplifying the duties of app developers. At the same time it carefully takes into account the concurrency issues associated with shared resource access, thus making sensing un-intrusive and minimizing the related impact on the perceived quality of user experience. MoST is a general-purpose library, which can be integrated also in other smartphone-oriented projects; in ParticipAct, we use it to effectively implement all the passive MCS sensing actions.

**Figure 3:**
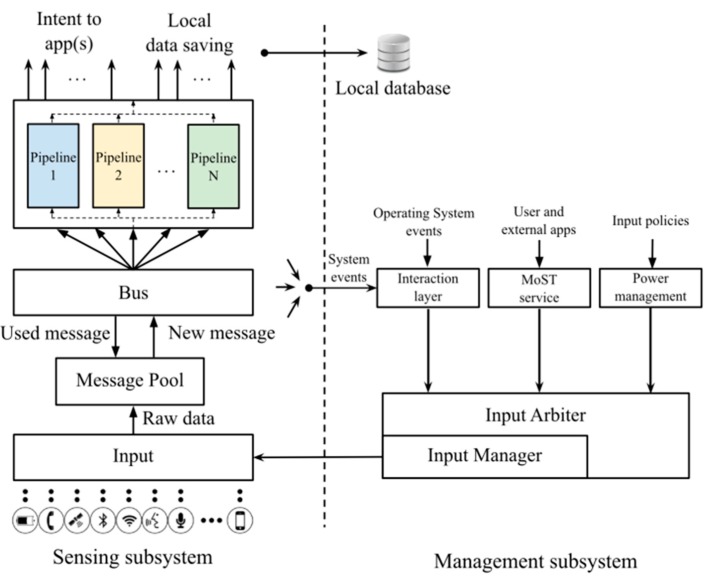
The MoST Architecture.

The MoST architecture is based on two primary building blocks: Inputs and Pipelines. Inputs are any physical or logical sources of sensing data (e.g., accelerometer, gyroscope, GPS, app networking statistics, battery level), while Pipelines are components that receive, process, and fuse sensed data collected from one or more Inputs by forwarding resulting data to client applications. In its turn, MoST consists of two main subsystems (Figure 3): the Sensing subsystem and the Management subsystem. The Sensing subsystem has a two-layered architecture and manages all aspects of sensing, from accessing Inputs, to wrapping them into easy-to-manage local objects that are dispatched to Pipelines. Pipelines then forward their results to client applications. The Management subsystem, instead, drives and commands the configuration/management of the sensing process, by providing an entry point to external apps to request MoST services, by resolving concurrency issues for non-shareable resources (e.g., the microphone cannot be physically used by MoST during a phone call), and by controlling power management. For more details on MoST, we refer the interested readers to [11].

### 3.3. The ParticipAct Server Architecture

**Figure 4:**
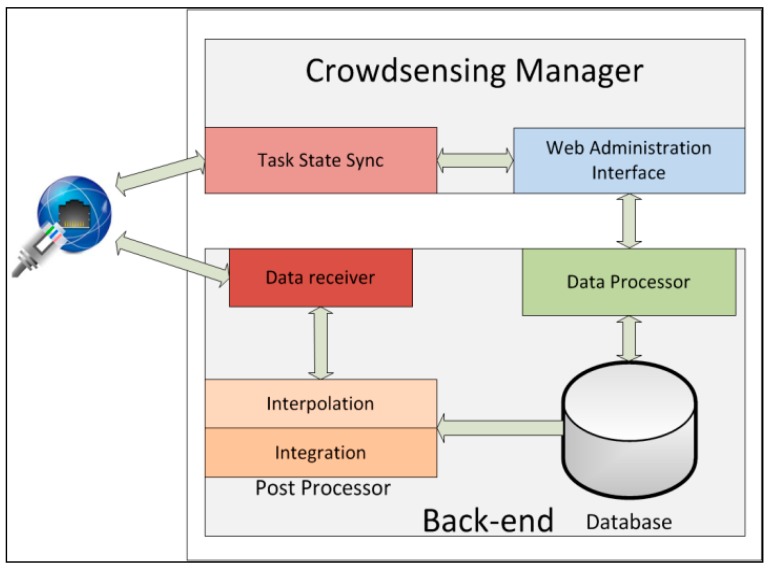
The server-side architecture of ParticipAct.

The ParticipAct server side provides management, storage, and analysis of crowdsensed data. At the highest level it comprises two main parts, as shown in Figure 4: the Back-end and the Crowdsensing Manager. The Back-end takes care of receiving, storing, and processing sensed data, while the Crowdsensing Manager provides the administrative interface to design, assign, and deploy sensing tasks.

In a more detailed view, the Back-end consists of three macro-components: Data Receiver, Post processor, and Data Processor. Data Receiver receives data from clients (namely, from Data Uploading components) via a Representational State Transfer (REST) Application Programming Interface (API) [12]. Data Receiver acknowledges each received data to allow data removal from the local database at the client side. Data are then cleaned up and prepared for long-term storage by Post-processor components, namely Interpolation and Integration. Interpolation improves data collection by filling in missing data points that can be inferred with sufficient accuracy. A notable case is geolocation data that on Android devices are available via the Google geolocation API, which dynamically switches between different techniques to infer user position (*i.e.*, GPS, Wi-Fi, and cellular 3G); this causes location accuracy to range and suddenly change from few meters (e.g., for GPS) to thousands of meters (e.g., for cellular 3G only). Interpolation substitutes data outliers, whose accuracy is significantly worse than the ones of temporally close data points, by substituting them with a simple linear interpolation of the more accurate data points; more sophisticated interpolation algorithms are simply pluggable in the ParticipAct architecture, but they are out of the scope of this specific paper. Integration, instead, aims at aggregating data in time and space. It collapses all the data, of any type, collected in the same 5 min window in a single row to enable time-based indexing of all sensed data and to successively speed-up the execution of temporal queries. It also aggregates data in space by creating a geographical view of sensed data, and storing it in a Geographic Information System database (GIS) for spatial querying. Finally, Data Processor exploits those time-based and space-based views to tailor user profiles for fast identification of users who are more likely to successfully execute a task according to ParticipAct assignment policies, as detailed in Section 3.2.2. Data Processor is also responsible for determining points (incentive mechanisms) to assign to every user for each task. As described in Section 4.2, each user receives different points based on her reputation; points are determined before creating the campaign and calculated on each user profile; each user will see on her own smartphone the points that will be earned after the successful completion of the currently considered task.

**Figure 5:**
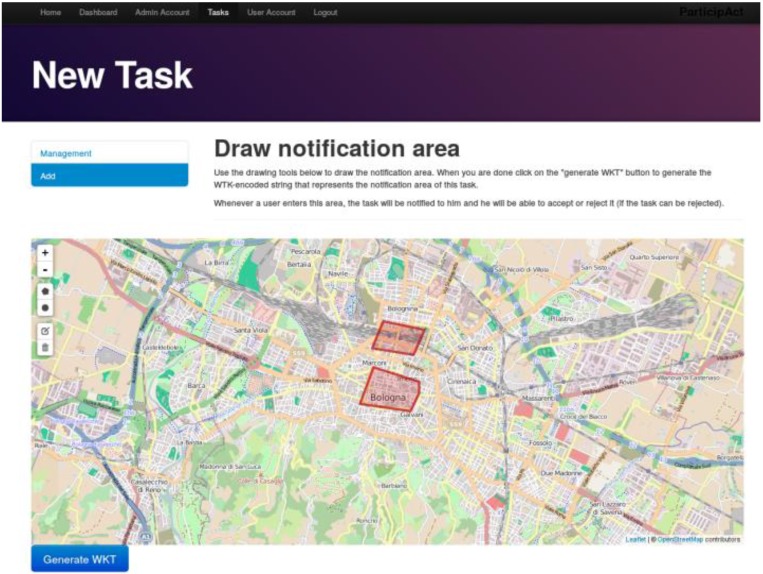
Screen capture of the ParticipAct Web Administration Interface. This figure shows the interactive page that allows to define the geo-notification area of a task.

Crowdsensing Manager is the administrator-facing part of ParticipAct. Web Administration Interface (Figure 5) allows smart city managers to interact with the ParticipAct platform and supports full-administration of the whole MCS process, including management of user profiles, design and assignment of tasks, and data review. A core function of the Web Administration Interface is its ability of tapping into results provided by the Data Processor, in order to automatically assign tasks to users who are more likely to successfully execute them. Task State Sync, instead, is in charge of keeping task state synchronized between clients and the server by pushing new tasks on designated clients and receiving all state change updates (e.g., task accepted/refused and task completed with success/failure). Among the several server functionalities supported in ParticipAct, in the next two sub-sections, we present two core features that represent hard technical challenges in the practical deployment of a real-world and widely adopted MCS platform: data transport and task assignment.

#### 3.3.1. Data Transport

An essential feature of a crowdsensing system is power-efficient, secure, and reliable data transport from clients to the long-term storage hosted on the back-end. Power efficiency is achieved on the client side (see also Section 4.1) by batching data transfers to minimize the number of times that network interfaces have to turn on to transmit data. Another important factor to reduce power consumption is limiting the amount of data that is actually transferred. To achieve this, ParticipAct serializes data bulks by using the highly efficient protobuf format [13], which significantly reduces the CPU consumption and data memory footprint if compared with native Java serialization and verbose serialization formats such as eXtensible Markup Language (XML) and also JavaScript Object Notation (JSON). Moreover, sensed data has often a low entropy (e.g., geolocation data usually differ in a limited way in a short time-span), which makes them highly compressible. ParticipAct exploits this aspect by having clients compressing all outgoing data with the lightweight—yet power-effective—gzip algorithm [14].

As regards security, authentication is provided by enforcing the usage of HTTP Basic Authentication for all user requests [15]. Data integrity and confidentiality is guaranteed by industrial standards for encryption: all REST and Web requests are available only via HTTPS over TLSv1 [16]. Moreover, HTTPS strengthens HTTP Basic Authentication, which by itself is a weak authentication mechanism prone to network sniffing attacks, by guaranteeing that user names and passwords are never sent in plaintext over the Internet. Finally, ParticipAct achieves reliability with a two-phase commit protocol to grant that all data are transferred from clients and stored at the back-end.

#### 3.3.2. Task Assignment

MCS task assignment is defined as the process of identifying the users who are more likely to accept and complete a task based on profiling, applicable context, and for example history of daily movements. Efficient task assignment is widely recognized to be a central key performance indicator for state-of-the-art MCS platforms, being the most crucial factor in achieving good MCS results by minimizing the resources consumed for users’ incentivizing. For these motivations, we have concentrated significant research efforts within the ParticipAct project to design and dynamically manage task assignment policies, by including articulated and original task management solutions and by devoting relevant efforts to assignment policy assessment and evaluation. Given the relevance and strong originality of this aspect, we have decided to dedicate the following Section 3.4 to go into in-depth technical details about the ParticipAct solutions for task assignment, as well as to have an in-depth discussion about lessons learnt, in particular in terms of acceptance, completion rates, and times of assigned tasks (either geo- or non-geo-notified), by considering tasks with different levels of complexity and duration.

### 3.4. The ParticipAct Task Assignment Policies

ParticipAct enables effective scheduling of geo-executed tasks through four main different policies, specifically designed and optimized for MCS campaigns, namely, *random*, *recency*, *frequency*, and *dbscan*, detailed in the following. Additional task assignment policies are easily pluggable in the platform via a related dynamic API.

The *random* policy selects a random subset of all available users, regardless of their position history, based on the user ratio parameter, which is defined as the percentage of all available users to be assigned to the task, from 0% up to 100%. It is an uninformed policy and we introduce it as the baseline solution, primarily to be used for the sake of comparison with the other more aware and informed task assignment policies.

The *recency* policy prioritizes the assignment of tasks to users who have been recently in the geo-execution area. This policy relies on the assumption that those users may return in the same area in their everyday commuting routine. Moreover, the *recency* policy ranks all potential candidates according to how recently they have been in the geo-execution area, from the most to the least recent. Similarly to the *random* policy, it may be configured with user ratios from 0% up to 100%, defined as the portion of candidates (starting from higher ranked ones) to select for an active role in the MCS campaign.

The *frequency* policy assumes that the people who visited more frequently the geo-execution area of the considered task are the best candidates to select; in other words, this policy implicitly assumes that those users usually stay or regularly attend the area. In particular, it selects users who have been in the target area in the past and ranks them according to the time that they spent there compared to the time spent in other places. In addition, as for *recency*, this policy supports user ratio setting in order to further limit the number of assigned candidates.

The *dbscan* policy uses the Density-Based Spatial Clustering of Applications with Noise (DBSCAN) algorithm to cluster past user location traces [17]. DBSCAN is a density-based clustering algorithm based on the idea that the density of points inside a cluster is much higher than that of the points outside the cluster, and that the density of points outside a cluster is much lower that the density of any other cluster. DBSCAN has several properties that make it well-suited for the problem of MCS task assignment: it does not require knowing the number of clusters to be determined *a priori*, it can detect arbitrarily-shaped clusters, it is robust to noise and outliers, and it is optimized to run on GIS-enabled databases. Our *dbscan* policy runs the DBSCAN algorithm over all past user positions and clusters those users who actually spend a sizeable amount of time in the target geo-execution area as potential candidates. Then, it selects users in a cluster that intersects the geo-execution area. Like all other policies, *dbscan* allows selecting a proper user ratio setting; however, differently from *recency* and *frequency*, since *dbscan* does not provide a ranking, the *dbscan* policy randomly determines the final set of selected users.

Note that all the above task assignment policies are sensitive to the size of location history used. In our work, we have decided to consider only a limited time window for the geolocation history of the last days before the considered task starts for each user (default configuration in ParticipAct is two weeks). If we consider all the history of a user, we can select, with higher probability, users who have changed routines and are not capable to complete that kind of task. Anyway, it is possible to modify that window size or even indicate specific hours of the day and/or specific days of the week in order to determine, with a finer grained approach, users who can complete tasks in a more specific time window.

One primary lesson learnt from our large-scale ParticipAct experience (and the associated in-the-field deployment of MCS campaigns involving large sets of users) is that the choice of the task assignment policy has a relevant impact on MCS results and efficiency. Therefore, in the experimental results section (Section 5), we will devote wide space to extensively report about our original efficiency metrics to assess the performance of task assignment policies in MCS campaigns and the associated performance indicators/lessons learnt deriving from our in-the-field measurements.

## 4. Users’ Involvement in ParticipAct: Gamification and Task Co-Creation

An original functionality of the most recent releases of the ParticipAct platform is the support of novel gamification and task co-creation features, with the primary goal to maintain a high level of participation and interest among the involved users. With the term gamification, we intend here the use of game design elements capable of fostering user engagement in context external to games, such as competition, success, and reward. By following these concepts, we have implemented in ParticipAct a general gaming framework based on three core elements, namely, reputation, ranking, and badges, in order to qualitatively and quantitatively experiment and evaluate the positive effects of the gamification approach in the specific context of MCS campaigns.

### 4.1. Reputation

Inside ParticipAct, we have implemented the concept of reputation with the primary goal of expressing and quantifying the reliability of a user towards a particular kind of task. In fact, every user has a reputation value for each specific type of action that she can execute. This reputation value summarizes the ability, and, consequently, the probability, that a user can complete that particular request, which is most relevant in managing the effectiveness and efficiency of MCS campaigns. This value evolves over time depending on the type of interaction between the associated user and the ParticipAct platform. In particular, the reputation value will increase every time a user completes with success a task containing that particular action and decrease every time a user will not complete a task that the user had previously accepted to complete. The overall user reputation will also vary, but in a slower and weaker way, when a user simply accepts or refuses any type of task, independently to the result of its execution (either completed successfully or failed). The rationale is in motivating users to accept a high number of tasks but, even more, not to fail in executing the accepted tasks. From the perspective of user-side awareness and visibility, the ParticipAct smartphone app shows the own current level of reputation (overall and for each type of action) to give user an immediate feedback on her MCS level of engagement.

### 4.2. Ranking and Points

Starting from reputation, the ParticipAct admin (e.g., a smart city manager organizing and optimizing MCS campaigns) can specify a policy for points/incentives assignment. The ParticipAct architecture is structured to simplify the addition of newly defined incentive policies at provisioning time, with no need to suspend or re-start the associated MCS campaign; different policies can be concurrently enforced for different situations and/or different tasks.

In particular, our ParticipAct server implements four different incentive policies based on two main concepts: reputation and level. As already stated, in ParticipAct we intend for reputation the direct ability and reliability of a user to complete a specific kind of action; reputation has a value between 0 and 100. For *level,* instead, we mean a mapping of the user reputation inside the 60–120 range for assigned points; we made this choice in order to limit diversity in points between users and to assign some non-zero points to users with still no reputation for a specific action. The incentive policies implemented in ParticipAct are four:

- *Average level*—the number of points to assign to a user is determined using the average value of levels of each kind of actions that compose the task;
- *Average reputation*—like the previous policy, but directly using the reputation value:
- *Sum level*—the number of points to assign to a user is calculated using the sum of the levels of each kind of actions that compose the task;
- *Sum reputation*—like the previous policy but directly using the reputation value.

Gamification experiences in other fields have demonstrated that users acquire points incentivized by the competing desire of climbing the leaderboard. Points are scarcely effective without a comparison term, so we have implemented a ParticipAct functionality that is capable to track position inside the leaderboard for every user and to show the situation on the participant smartphone. It is also possible to find friends through standard and widespread social networks and showing a personalized leaderboard reduced to friends. An additional feature that we have decided to implement is the aging of points: points automatically decrease over time; in this way, it is possible to remix leaderboard and keep users on the bottom alive, with the easier possibility to climb the board in the next MCS campaigns.

### 4.3. Badges

As a further incentive, we have implemented in ParticipAct the concept of unlockable badges. They may associate with a given task: when this task is completed with success, a badge appears on the completing user’s smartphone. Or they may associate with an action type: after successfully completing a task with a specific action type, a type-related badge is prompted on the user smartphone. Badges for a given task have usually short-term validity and usage; for long-term influence, a badge associated with a series of tasks, completed with success and with specific action types, is most appropriate.

### 4.4. Task Creation

To involve users even more deeply in ParticipAct MCS campaigns, we have also decided to give them the capability of new task definition. Users can access ParticipAct platform functionality similar to MCS administrators: they can define a task campaign involving other users by accessing the tools provided ParticipAct for this purpose. In this way, users are directly involved in the process of data gathering; most important, involvement can increase while stimulating crowd-oriented creativity; moreover, this encourages final users to understand better the capabilities of the platform and to be aware of its possible limitations.

Users can define new tasks either through the smartphone app or through the ParticipAct Web server and, by using a guided and controlled process, can submit new tasks to the ParticipAct administrator. The user-generated tasks, before starting the flow seen in Figure 1, undertake a new sub-flow where they stay in an idle state while waiting for the administrator to approve them. If approved, points are assigned to the user that proposed the new task and the task is prompted to other users, thus beginning its associated task flow. In conjunction with the support of social relationships and consideration of users’ friendships, it is possible to suggest administrators to assign tasks to task creators’ friends, thus stimulating direct cooperation/competition with friends and increasing active participation.

## 5. Experimental Results and Lessons Learnt

We claim that the ParticipAct experience is interesting and unique inside the field of MCS for different motivations. First, it is a real-world deployment of a complete MCS platform with interesting features. Second, it has allowed to collect a complete and very large dataset of crowdsensed information, involving a large number of participants for a very long time duration. Third, the thorough analysis of those collected data has originally allowed us to point out lessons learnt and solution guidelines to maximize the ParticipAct platform strengths and to define best practices for future MCS platforms. This section first defines novel and suitable metrics to compare task assignment policies for MCS campaigns, then presents a quantitative assessment of our assignment policies for geo-executed tasks, and finally shows a comparison of task acceptance and completion rates/times for different kind of tasks.

### 5.1. MCS Evaluation Metrics

A widespread and internationally recognized consensus on the evaluation metrics for the analysis of MCS campaign performance has still to be reached in the research community, because of the lack of wide MCS datasets with real-world results about task assignment policies as well as task acceptance and completion. This paper also provides a contribution to the MCS community of researchers by proposing novel and usable MCS evaluation metrics.

Let us focus first on the MCS metrics that can assess the effectiveness of task assignment policies. The number of assigned users is defined as how many candidate users a given policy assigns a specific task. Precision measures the percentage of success of a given policy, namely, whether selected users where the ones who actually executed the assigned task successfully. For this evaluation, True Positives (TP) users are the ones selected by a policy and who actually carried out the task, while False Positives (FP) are the users selected by a policy but not executing the task. We define precision as the ratio between TP and TP + FP. Moreover, True Negative (TN) users are the ones who have not been selected and did not execute the task, while False Negatives (FN) the users who have not been selected but did execute the task anyway. Accuracy is a percentage and accounts for the proportion of true results (both true positives and true negatives) in the population. More formally, accuracy is the ratio between TP + TN and TP + FP + TN + FN, that quantifies how good is each policy in correctly classifying user behavior and predicting whether they will (TP) or will not (TN) execute a task. Finally, we originally introduce some simple metrics to evaluate task acceptance and completion. Acceptance rate and completion rate represent the percentages of users (evaluated over all involved people) who, respectively, accepted and completed the task. Similarly, acceptance and completion times represent the duration of the interval required to, respectively, accept and complete a task.

### 5.2. Evaluation of ParticipAct Task Assignment Policies

The experimental assessment of a large scale crowdsensing system in a realistic scenario poses significant social, technical, and logistic challenges. In a long-running effort to test ParticipAct, we are currently maintaining a large deployment that involves 173 volunteers, all of them students of University of Bologna from different courses and year, that are attending on either the Bologna campus (123 students) or Cesena campus (50 students). Although, as for other similar experiments, it is an open question if obtained results could hold in a more general scenario, we believe the ParticipAct dataset is large enough (in time and space) to draw some first important observations, rather realistic for urban setting scenarios. In fact, it is important to underline that Bologna and Cesena university campuses are not self-contained: they comprise several dozens of different buildings spread over these two metropolitan areas. For this reason, volunteers’ path and behavior are not limited to a specific area but related to the whole urban territory that coincides with the same area of all citizens living in the same smart city.

Of course, we cannot present all the tests we have done in the first ParticipAct period, because we have triggered many different campaigns. In this section, we present a quantitative assessment of our assignment policies for geo-executed tasks. In the next section, we present an analysis of acceptance ratio for different types of tasks proposed to our volunteers, and we compare differences of acceptance based on notification type (namely, geo-notified or not).

As regards deployment aspects, we provided each volunteer with a Samsung I8190 S III Mini with pre-installed ParticipAct client. The ParticipAct client reports user geolocation every 180 s, thus allowing us to have very precise mobility traces. On the server side, ParticipAct was developed as a Spring MVC web application hosted on Apache Tomcat 7.0. The server hosting the web application uses an Intel i5 3210 M 2.5 GHz CPU, with that 8 GB of RAM, and is connected via a 100 Mbit connection to the server hosting the database that stores all crowdsensed data. The database server uses an Intel Xeon E31240 3.3 GHz CPU and 8 GB of RAM, and runs PostgreSQL v9.1 DBMS, that has been enhanced with PostGIS v1.5 to run geographical queries. All geolocation traces of users have been stored in a GIS-enabled table to allow fast geographical queries.

**Figure 6:**
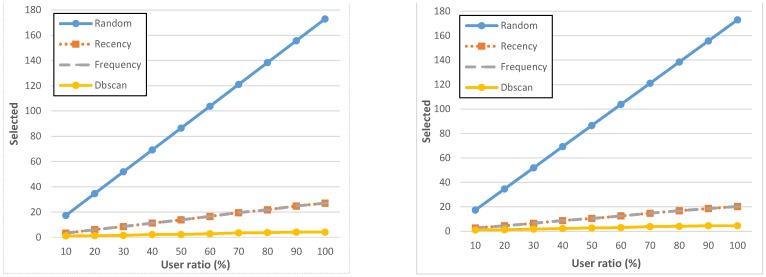
Number of candidates selected by each policy in case of geo-notified (**left**) and non-geo-notified (**right**) tasks.

Figure 6 shows the number of candidate users selected by each policy (over the total of 173 volunteers) for various geo-executed tasks; we considered four geo-notified tasks (graphs on the left) and four only geo-executed, but non-geo-notified (graphs on the right). For all tasks, we have analyzed the ParticipAct collected data to understand which performance values would have been achieved if users had been selected by different policies and by using different user ratios in the range [10%, 100%]. In the following, all results represent average values over, respectively, the four geo-notified and the four non-geo-notified tasks. Because they were executed by the same population and were associated to urban areas with similar characteristics, they have comparable completion/failure rates.

As expected, the random policy shows the worst performance. Recency, frequency, and dbscan policies always select about twenty users or less, with dbscan selecting very few users compared to recency and frequency, always below five users as the average value. These policies (except the random one) significantly limit the number of users assigned to a task by not affecting the final success of the MCS campaign, thus reducing the workload of users and the “process costs”, e.g., in terms of resource-consuming incentives to be attributed to users completing the assignment successfully.

**Figure 7:**
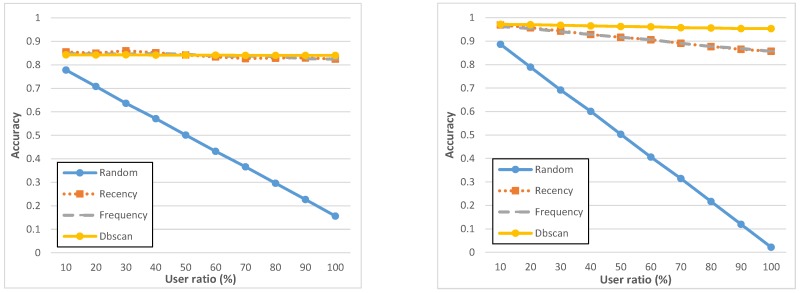
Accuracy on geo-notified (**left**) and non-geo-notified (**right**) tasks.

**Figure 8:**
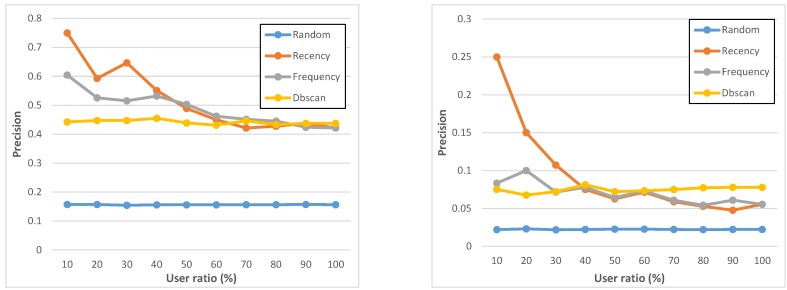
Precision of geo-notified (**left**) and non-geo-notified (**right**) tasks.

Figure 7 and Figure 8 report precision and accuracy performance results, by showing that all of them have specific strengths and weaknesses, depending on deployment conditions and MCS campaign-specific constraints, for instance about user ratio. The *recency* and *frequency* policies perform slightly similar and it is important to state that the list of assigned users produced by *recency* and *frequency* contain the same set of users, but ranked differently: this is why, with 100% user ratio, they reach exactly the same results. At the same time, let us also rapidly note that some (minor) oscillations in Figure 8 are due to the fact that, notwithstanding the good number of users involved in the ParticipAct MCS campaigns, the cardinality of participants is still sufficiently low to exhibit stochastic fluctuations, especially for *recency*. If compared with *recency* and *frequency*, the *dbscan* policy has a higher accuracy and shows overall a very stable behavior for all considered metrics. Most important, it obtains those results with a very low number of assigned users, thus confirming DBSCAN ability to capture and cluster routinely user behaviors. Finally, a very important lesson learnt from our analysis is that geo-notification should be applied whenever possible: indeed, notifying potential candidates only when they enter the interested geo-execution area allows one to boost the task completion rate and, consequently, the associated precision. We believe that this is due to the fact that candidates who are notified beforehand (non-geo-notified) tend to forget completing the task as they reach the geo-execution area.

### 5.3. Task Acceptance and Completion Analysis

**Figure 9:**
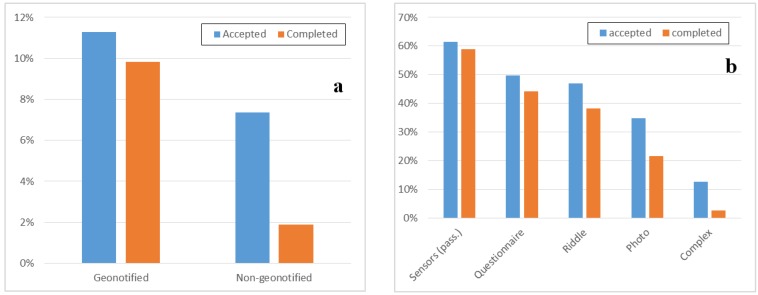
Acceptance (**a**) and completion (**b**) rate for different types of task.

We have learned from direct experience and in-the-field measurements that selecting the most appropriate assignment policy can lead to significantly better MCS results. Anyway, other non-negligible aspects affect the MCS campaign efficiency, such as the different modes with which users can be involved in participating to different kinds of tasks. To better understand and analyze the impact of different task types assignment based on context, we have thoroughly investigated this behavior through our huge dataset of historical data about task assignments. In particular, Figure 9 reports results on acceptance and completion for different kinds of tasks. Figure 9a shows the difference in geo-notified (on the left) and non-geo-notified (on the right) tasks; for this purpose, we considered eight tasks either geo-executed or non-geo-executed and they all have similar characteristics in term of task type, area of execution and notification, and duration. The reported results confirm that geo-notification allows improving acceptance and completion rates respectively by 52% and 423%. Figure 9b, instead, reports acceptance and completion rates for different types of tasks, from passive and more easy-to-complete (shorter and simpler) on the left, to more and more complex ones on the right. Users are more willing to accept and complete passive tasks (without need of user intervention, such as GPS monitoring) and simple active tasks (with limited actions). Instead, for complex task that aggregate multiple actions, users tend to be less willing to participate and complete them.

**Figure 10:**
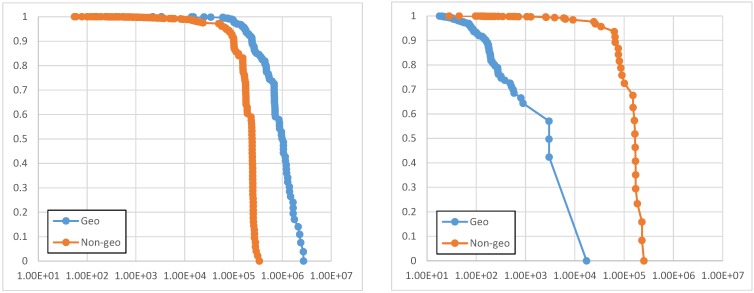
CCDF of acceptance (**a**) and completion (**b**) time (in seconds) for geo-notified and non-geo notified tasks.

Another relevant factor for crowdsensing campaigns management is the acceptance/completion rate dependency: in order to better understand this relationship and to originally contribute with real world data about this, our results analysis continues by showing the Complementary Cumulative Distribution Function (CCDF) for acceptance (Figure 10a) and completion (Figure 10b) times for geo-notified and non-geo-notified tasks, by considering the same tasks and users of Figure 9a. In particular, Figure 10b reports the latency for receiving crowdsensed data results (time interval between task creation and result reception). This performance indicator is useful to understand (and then confirm expectations from) crowd behaviors. In fact, as expected, acceptance time has demonstrated to be higher for geo-notified tasks for the motivation that, in this case, task notification is delayed until users enter the task notification area. The completion time, instead, is relatively lower in the case of geo-notified tasks because they are typically notified in the area and then completed by users right away, typically in less than 15 min. This confirms an expected behavior (however, in-the-field, real-world experimental results about that are original in the related literature) but is central for easy and effective improvement of the efficiency of crowdsensing campaigns through automated frameworks, such as ours, capable of selecting dynamically the most proper scheduling and priority strategies in order to optimize domain-specific constraints on economic budget and other key performance indicators.

**Figure 11:**
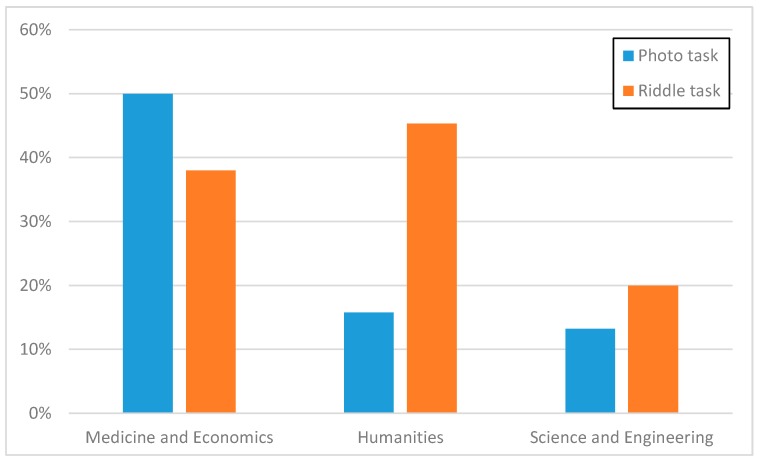
Ratio of success by users’ university course.

Let us note that the experimental data collected in ParticipAct are very rich and well-suited for a number of possible aims and further analysis, e.g., with the goal of user profiling. Just to provide an example and for the sake of briefness, let us mention the fact that we worked on aggregating information based on the different Schools where ParticipAct volunteers are enrolled. Figure 11 shows users’ responses to two different sets of tasks. The first set relates to photo tasks where users are required to take pictures of different city venues. The second set is a collection of riddle tasks where users have to reply by guessing a word that has connections with all other proposed words. As we can see from Figure 11, riddle tasks have demonstrated much higher completion rates among Humanities students, by confirming the expected users’ profiling, while Medicine and Economics students seem to be more interested in photo tasks, probably also due to the fact that their Schools are located in the city center. Finally, our profiling analysis has quantitatively confirmed to us that we have to carefully consider the consolidation of our collected results by taking into account some possible bias effects stemming from the fact that that the majority of our volunteers (135 out of 170) are from the School of Engineering and Architecture: different dynamic weights are under consideration in order to smoothen these bias effects, with the general idea that wider population generally means wider statistical validity but with the need of correction metrics based on population diversity and other factors, such as usually lower motivation in wider sets of involved volunteers.

### 5.4. Participation

Here, we start by presenting a performance indicator aimed to assess the involvement degree of our volunteers; then, we focus on the gamification features presented in Section 4, by reporting some quantitative results capable of quantitatively pointing out about their effects in real-world deployment scenarios.

**Figure 12:**
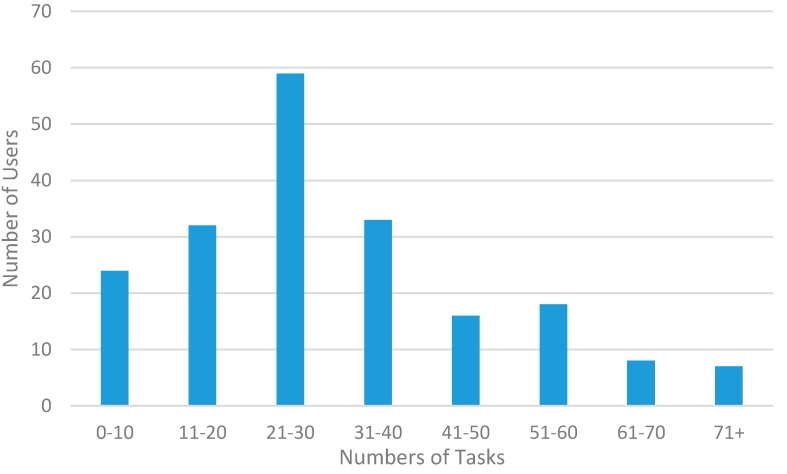
Number of tasks completed by users.

Figure 12 shows the level of participation of our volunteers to the proposed crowdsensing campaigns by displaying how many users completed how many tasks. As expected, the distribution has a bell shape with a long tail: Most users have completed around 30 tasks; a good number of volunteers completed more tasks, from 31 to 71+; finally, some users have completed more than 100 tasks by exhibiting much more interest in ParticipAct involvement than average users; no one completed all the proposed tasks.

By focusing on gamification, we have compared users’ involvement before and after the introduction of these features, and tried to evaluate the impact of the employed gamification technique in fostering participation. We first noted that, after a boost of participation in the first starting months of the ParticipAct experiment, the users were less prone to accept new tasks: the rate of acceptance of a new task settles to 15.8% and the rate of users ignoring new tasks settles to 78.5%, thus showing a symptom of limited interest towards our MCS campaigns. After introducing the presented gamification features, ParticipAct acceptance rate is returned back to significantly better levels, such as 25.5%, after two months from the new version deployment; in our comparison, we used simple routinely tasks with comparable completion complexity in order to avoid effects related to differences in task nature.

**Figure 13:**
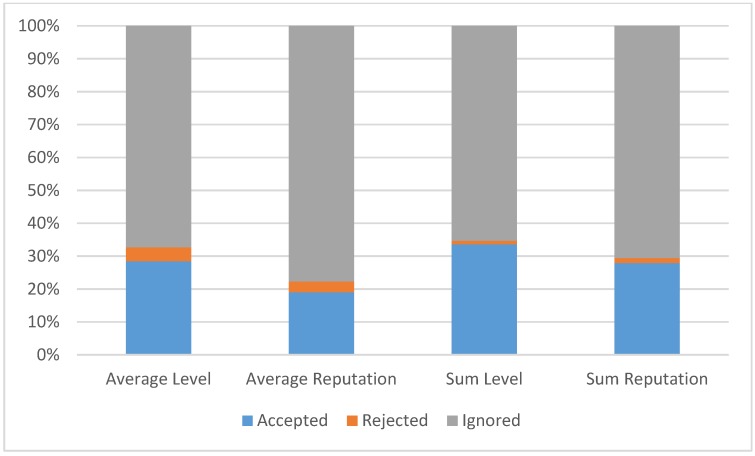
Point assignment strategies comparison.

Another important element, not covered by significant in-the-field performance results in the related MCS literature, is exactly how much incentive strategies (e.g., points rewarding) can affect participation. Figure 13 shows that our in-the-field real-world experience has demonstrated that “level” strategies have much higher impact and are more effective in terms of users’ response. In particular, via analysis results and users’ questionnaires, we have found that the main reason of this more effective impact is in the fact that they are “more generous” and “more rapid” ways for users to climb their leaderboard. It is also interesting to notice that “level” strategies should not be abused because they can also have the negative side effect to extend the difference between the first and last users in the leaderboard, with a non-negligible negative effect on de-incentivizing users who are currently low ranked.

**Figure 14:**
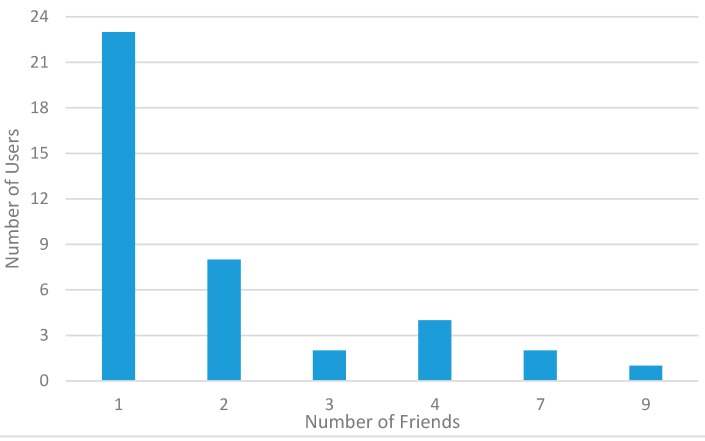
Number of users with a specific number of friends.

About social relationships and friendship, Figure 14 reports experimental data on how many friends the considered ParticipAct volunteers have among the ParticipAct users themselves. We can notice that, notwithstanding the relatively large community of ParticipAct users, they are structured into friendship groups of limited size: most users have only one friend involved in the MCS campaign, and only one has nine friends. This graph shows that our user base is distributed among student population with limited group size (if compared with the total number of potential participants). This situation roughly reflects the usual case for citizen volunteers in a large city, where population is organized in small groups (if compared to total population) if clusterized, for example, on the places they attend. In the same way, ParticipAct users are organized in small groups from their graduation courses from different schools, which are typically located in different parts of the city of Bologna.

**Figure 15:**
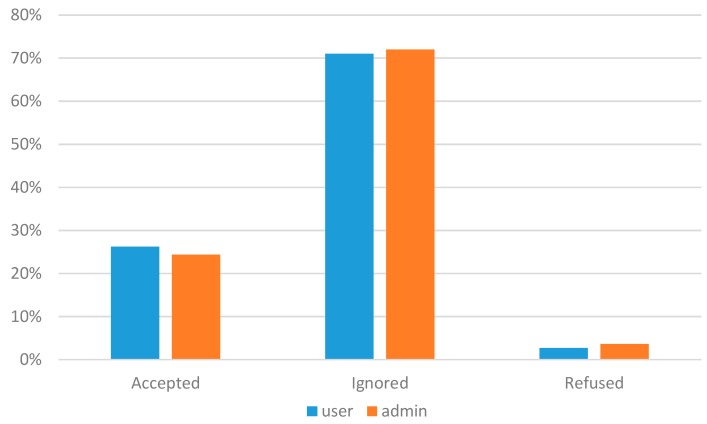
Participation in administrator’s task and user’s task.

During the last two months, we have also assessed and evaluated the ability and the effects of ParticipAct users to create new tasks. In this time span, we observed a growing users’ involvement and a grown interest in being aware of how the ParticipAct platform runs and which facilities it makes available for app developers on top of it. User-generated tasks were 30 in two months, while, in the same period, the number of tasks created by ParticipAct administrators were 29. Via questionnaires and interviews, we have understood that users are interested by this new functionality, both because they feel more than they can contribute to the overall MCS campaigns and because they can understand better the technical facilities/capabilities of the ParticipAct platform. Interestingly, after that, they have also relevantly increased their active behavior of suggesting improvements to the task definition feature and to the whole platform, primarily in order to improve usability. Figure 15 shows the behavior of users against tasks created by either platform administrators or “regular” users in the same time span: the reported results show a generally higher interest for tasks created by users, probably because of a more alike way of thinking, because of similar interests, and because of insider effects related to participation incentivizing within small communities (e.g., individuals solicited to accept task assignments by their friends). Anyway, the evident and measurable result is that community-based task creation is demonstrating itself to be a valuable way to keep the users’ interest in actively participating in MCS campaigns high.

## 6. Related Work

Interest in crowdsensing has seen a tremendous growth in the recent years, in both industrial and academic research, thus promoting the development of several relevant MCS platforms. A complete crowdsensing system covers several different research topics, including signal processing, machine learning, distributed systems, and social sciences; accordingly, there are several research efforts in the literature focused on each of these aspects, considering often each of them separately. A significant original aspect of the ParticipAct approach is that our platform tries to tackle the whole stack of technical and social problems related to MCS, which poses evidently significant challenges. In the following, without ambition of completeness, we overview and discuss some of the most relevant efforts in the area, by comparing them against ParticipAct. We start presenting the MCS solutions that are more distant from ParticipAct, either for application-specific purposes rather than general-purposes or because it is only including a subset of all needed MCS functions; then, we terminate the section with those closer to our platform in both goals and supported facilities. The section is concluded with a brief overview of the very few and seminal MCS living labs focused on MCS dataset collection, whose approach is very close to our project.

Starting with the solutions focusing on task assignment management, Ohmage is a healthcare-oriented system that exploits smartphones to collect both passively and actively information about users [18]. Ohmage system architecture, similarly to ParticipAct, comprises an Android app to collect data and a back-end that allows to administer data requests and then to visualize and analyze the collected data. Differently from ParticipAct, Ohmage has no means to tie data requests to a specific geographic area, thus reducing its usefulness for smart city scenarios that could require users to be in a specific place to effectively execute a task.

Vita is a system that stresses the relevance of providing crowdsensing as a service integrated with usual software and supports sensing task assignment based on user profiles [19]. To achieve the first goal, Vita relies on BPEL4PEOPLE, a Business Process Execution Language extension that enables orchestration of human-driven sensing tasks within the Web Services technology ecosystem [20]. To achieve the second goal, Vita assigns tasks and users to a so-called “social vector”, which is a concise representation of user resources and knowledge; the social vector is exploited to assign tasks to users whose profile suggests that they may be willing to accept those tasks and have enough resources to complete them successfully. While Vita provides a nice support for non-geo-executed tasks, it completely lacks support for advanced task assignment policies for geo-executed tasks based on user movement history.

Matador is a crowdsensing software that focuses on context awareness to optimize task assignment while minimizing battery consumption [21]. In particular, Matador assumes that a task is defined by geographical and temporal dimensions, and should be assigned to users that are within the given geographical area in the given time window; to that purpose, Matador drives the sampling time of user positions to minimize battery consumption, by dynamically switching between network-based geolocation (power-efficient but inaccurate) and GPS (power-hungry but more accurate). ParticipAct adopts a more proactive approach and, differently from Matador, allows us to assign geo-executed tasks to volunteers based on their past mobility history, without assuming constant communications at runtime but only requiring lightweight and infrequent geo-localization sampling at client devices.

USense is a middleware for community sensing that strongly decouples users collecting data and managers requiring MCS data: managers specify which kind of data they need and USense matches them with people meeting the requirements [22]. A notable feature of USense is its flexible policies for smartphone sensors duty cycling, which enables the reduction of battery consumption for sensing activities. Similarly to USense, also the MoST sensing core of ParticipAct supports duty cycling of passive sensing activities.

The Medusa framework focuses on algorithms to define crowdsensing tasks [23]. Medusa is based on a domain-specific programming language that provides high-level abstractions to specifically define crowdsensing tasks, and employs a distributed system that coordinates the execution of those tasks between smartphones and a cluster in the cloud. By providing programming abstractions for the definition of the tasks, Medusa is complementary to our ParticipAct work, but at the current stage of its implementation, it lacks task assignment management support of geo-executed tasks. In addition, similarly to Matador and Vita, it also lacks the signal processing and machine learning support to automatically collect high-level inferences about user activities.

Finally, other research efforts, complementary to ParticipAct, were directed towards energy-/cost-efficient crowdsensing. In [24], authors presented an energy-efficient mobile crowdsensing framework where data transmissions of collected data were operated during a phone call. By this method, operations requested for transfer were shared with phone calls, and that allowed a significant savings in energy. In CrowdTasker [25], instead, the same authors focused on task assignment using incentives with the objective to maximize the sensing task coverage for a specific location while operating under budget constraints.

As regards large experiments aimed to build large crowdsensing datasets, the ParticipAct collected dataset is a relevant sample (and the basis the model) for the mobility of students in the Emilia Romagna region in Italy. The data collection campaign, which is still running, began in December 2013. Our ParticipAct smartphone client, among other things, tracks the location of its device by using the Google location APIs (by fusing GPS and WiFi Hot Spot coordinates) by using a sampling scan period of 150 s. Note also that the mobility in ParticipAct is unrestricted: users live in town or sub-urban areas; some of them commute daily by train, while others walk or move by bike; this unrestricted mobility is often a realistic feature not present and considered in other MCS experiments.

A dataset with technical characteristics similar to the ParticipAct one is the MDC Nokia dataset [26,27]. In MDC, the data were collected from 2009 to 2011, by involving 185 users in the Lake Geneva region (CH). The MDC users carried a Nokia N95 phone, with an application that periodically collected several local data, such as GPS, Bluetooth sightings, visited places, SMS, phone calls, and other sensor data. The sampling period for all the collected data (including GPS and Bluetooth traces) was 600 s in MDC, which is significantly larger than in ParticipAct. Furthermore, ParticipAct closely mimics a more realistic MCS scenario where users should be able to freely decide whether they accept a task or refuse it, while in MDC data collection is compulsory to all participants.

Let us rapidly and finally notice that, in the literature, there are some other platforms tested over real deployment environments, but most of them focus on collecting data from sensors without user involvement. What ParticipAct do is also to provide a platform for defining and proposing tasks associated to actions, both passive and active ones from the user’s point of view.

## 7. Conclusive Remarks and Directions of Future Work

In this paper, we have presented ParticipAct, our ongoing crowdsensing project at the University of Bologna that involves 173 students that are currently participating in a large-scale and long-running experiment of MCS campaigns. Even if the MCS topic has attracted much research interest recently, there is still lack of real-world large-scale MCS datasets and Living Labs able to truly verify any step in the whole MCS process, from mobility to task scheduling, from task acceptance to task completion. Hence, we strongly believe that the ParticipAct experience could pave the way to a new generation of effective, efficient, real-world, and highly scalable crowdsensing testbeds, with relevant lessons learnt towards the realization of significant MCS campaigns working as effective monitoring solutions for the Smarter Cities of our near future.

The encouraging results achieved so far within the ParticipAct project are stimulating our further research work in the field. In particular, we are primarily working along two research directions. On the one hand, we are realizing new smart city services and applications based on the use and specialization of the ParticipAct MCS platform. On the other hand, we are extending the ParticipAct task (co-)creation facility to support also the definition and instantiation of more complex task workflows, for instance, to let a participant slit her task into subtasks to delegate to friends, with possible hierarchical collection of sub-results at delegated participants.
